# Establishment and characterization of a rat model of scalp–cranial composite defect for multilayered tissue engineering

**DOI:** 10.1097/JS9.0000000000002108

**Published:** 2024-10-07

**Authors:** Yi Zhu, Ou Mei, Hui Zhang, Wulin You, Jiamin Zhong, Caralyn P. Collins, Guowei Shen, Changqi Luo, Xingye Wu, Jingjing Li, Yi Shu, Ya Wen, Hue H. Luu, Lewis L. Shi, Jiaming Fan, Tong-Chuan He, Guillermo A. Ameer, Cheng Sun, Liangyuan Wen, Russell R. Reid

**Affiliations:** a Molecular Oncology Laboratory, Department of Orthopedic Surgery and Rehabilitation Medicine, The University of Chicago Medical Center, Chicago, Illinois, USA; b Department of Orthopaedic Surgery, Beijing Hospital, National Center of Gerontology, Chinese Academy of Medical Sciences and Peking Union Medical College, Beijing; c Department of Orthopedics, Jiangxi Hospital of Traditional Chinese Medicine, Jiangxi University of Traditional Chinese Medicine, Nanchang; d The Breast Cancer Center, Chongqing University Cancer Hospital, Chongqing; e Department of Orthopaedic Surgery, Wuxi Hospital Affiliated to Nanjing University of Chinese Medicine, Wuxi; f Ministry of Education Key Laboratory of Diagnostic Medicine, and Department of Clinical Biochemistry, School of Laboratory Medicine, Chongqing Medical University, Chongqing, China; g Department of Mechanical Engineering, Northwestern University, Evanston, Illinois, USA; h Department of Orthopaedic Surgery, BenQ Medical Center, The Affiliated BenQ Hospital of Nanjing Medical University, Nanjing; i Department of Orthopaedic Surgery, Yibin Second People’s Hospital, Affiliated with West China School of Medicine, Yibin; j Department of Gastrointestinal Surgery, The First Affiliated Hospital of Chongqing Medical University, Chongqing; k Department of Oncology, The Affiliated Hospital of Shandong Second Medical University, Weifang; l Stem Cell Biology and Therapy Laboratory of the Pediatric Research Institute, The National Clinical Research Center for Child Health and Disorders, and Ministry of Education Key Laboratory of Child Development and Disorders, The Children’s Hospital of Chongqing Medical University, Chongqing; m School of Biomedical Engineering, Capital Medical University, Beijing, China; n Department of Biomedical Engineering Northwestern University, Evanston, IL; o Laboratory of Craniofacial Biology and Development, Section of Plastic and Reconstructive Surgery, Department of Surgery, The University of Chicago Medical Center, Chicago, IL; p Department of Surgery Feinberg School of Medicine, Chicago, IL; q Center for Advanced Regenerative Engineering, Northwestern University; Evanston, IL 60208, USA

## Introduction

Highlights

- Utilizing a 3D-printed wound obturator, the cranial–scalp composite defect model effectively slowed the scalp healing process and preserved the cranial defect, embodying the characteristics of a ‘chronic composite defect’.
- In parallel, an autologous reconstruction model was established as the positive control, which exhibited reproducible skin healing within 3 weeks with variable degrees of osseointegration, consistent with clinical practice.
- Both models provide a stable platform for subsequent research for composite tissue engineering and scaffold design and mechanistic studies of composite tissue healing.

Composite cranial defects, which affect multiple tissue types, pose significant challenges for reconstructive surgeons^1^. Many of these cases cannot undergo one-stage repair due to factors such as concurrent infections, polytrauma, ongoing radiation therapy, or prior cranioplasty failures, thereby leading to chronic defects. In recent decades, biomaterial-based cranial or scalp repair through tissue engineering has emerged as a focal point in research^2^. However, composite defect rodent models, particularly those that mirror chronic clinical conditions, are rarely reported. The primary challenge is the rapid rate of skin healing in rodents, which can culminate within 2 weeks.

**Figure 1:**
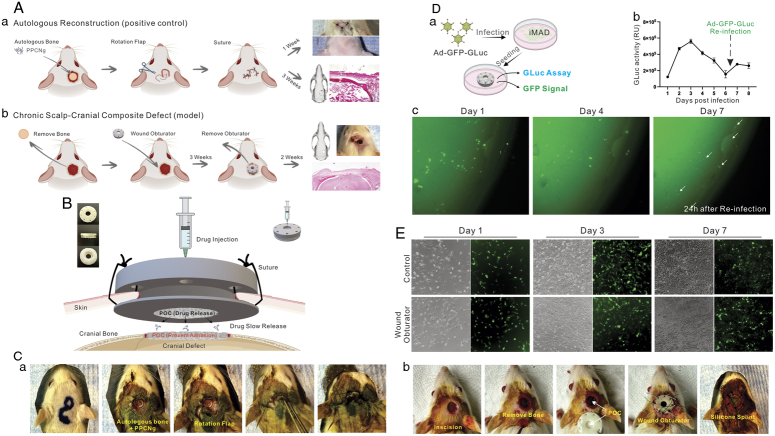
Study design, surgical procedures, and wound obturator *in vitro* assays. (A) Illustration of autologous reconstruction model (positive control) (*a*) and chronic scalp–cranial composite defect model (*b*). (B) Design of WO and its application in surgery. (C) Surgical procedures. In the positive control (autologous reconstruction) group, the excised bone was replaced as an autograft with 100 µl glycol citrate-co-*N*-isopropylacrylamide gelatin (PPCNg), and the overlying wound was reconstructed using a rotation flap (*a*). In the experimental group, a citrate-based polymer poly(octamethylene citrate) (POC) disc was implanted into the cranial defect to prevent adhesion between the dura mater and granulation tissue. The incision was further supplemented using either a wound obturator or a silicone splint (*b*). (D) Subconfluent iMAD cells were transduced with Ad-GFP-GLuc. Post-infection, cells were applied to WO submerged in a culture medium. After 6 days, the cell-laden WO was reinfected with Ad-GFP-GLuc (a). Both Gaussia luciferase activity (b) and GFP fluorescence (c) were monitored as indicators of cell viability. The white arrows indicate the reinfected cells. (E) Co-culture images of WO with iMADs. Viable cells were stained with Calcein-AM and imaged at the indicated time points. Representative results are shown.

In this study, we developed a clinically relevant, chronic composite cranial defect model in rats (Fig. 1A). The experimental model effectively slowed the scalp healing process and preserved the cranial defect, embodying the characteristics of a ‘chronic composite defect.’ The positive control exhibited reliable and reproducible healing through autologous reconstruction, mirroring current surgical practice.

## Materials and methods

The wound obturator utilized in the experimental group is depicted in Figure 1B. This device, crafted from resin xFLEX475-white (NEXA 3D) through 3-D printing, comprises two rings that securely anchor the scalp, while an inner cylindrical wall offers mechanical resistance against centripetal scalp growth. Cell viability assay and WO biocompatibility assay were performed as previously described^3,4^.

The protocols of this study were approved by the Institutional Animal Care and Use Committee (IACUC) of the University of Chicago (ACUP #71445). The work has been reported in line with the ARRIVE criteria^5^. Images of the surgical procedures are shown in Figure 1C. In the positive control (autologous reconstruction) group, the excised bone was replaced as an autograft with 100 µl of a biocompatible, thermosensitive polymer, polyethylene glycol citrate-co-*N*-isopropylacrylamide pre-mixed with gelatin (PPCNg)^6^, and the overlying wound was reconstructed using a rotation flap. Conversely, the negative control group was left with the bone and scalp defects untreated; the wound was merely wrapped in gauze to inhibit infection. For the experimental group, the incision was supplemented using either a standard silicone splint (Grace Bio-Labs) or a wound obturator to counteract the rapid initial self-healing of the scalp post-trauma. Three weeks post-initial surgery, the experimental group (WO group) underwent a secondary operation. Mimicking clinical conditions, granulation and connective tissues were meticulously excised to refresh the margins of bone and scalp. By this secondary method, the surgically debrided defects are primed for skin-bone tissue engineering via regenerative scaffold placement.

## Results

The biocompatibility assay demonstrated that Ad-GFP-Gluc infected iMAD cells could survive on the obturator for more than 7 days (Fig. 1D). Upon reinfection with Ad-GFP-Gluc, Gaussia luciferase activity was partially restored and emergent GFP signals were observed overnight. Furthermore, results from the cell-obturator co-culture assays revealed no significant differences in cell proliferation rates at days 3 and 7 (Fig. 1E), suggesting that the material used for 3-D printing of the wound obturator may exhibit little or no significant cytotoxicity.

**Figure 2:**
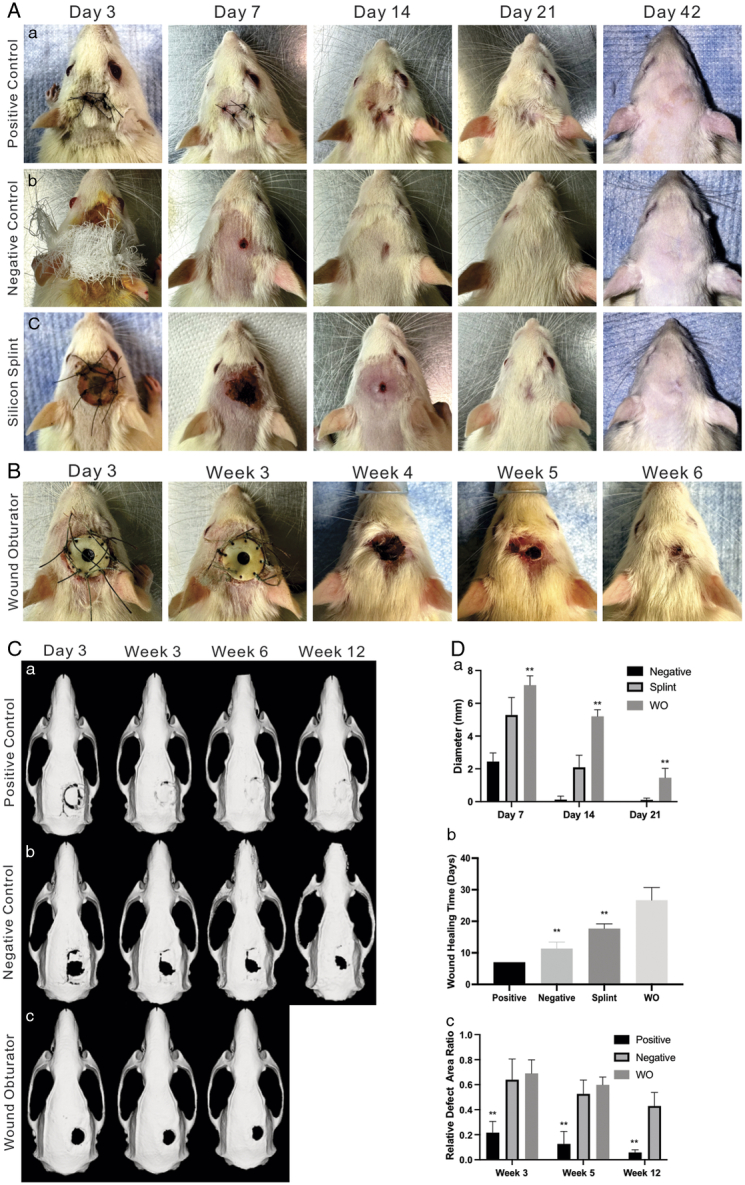
Scalp and cranial healing conditions at indicated time points. (A) Representative scalp healing images of the positive group (a), the negative group (b), the silicone splint group (c), and the wound obturator (WO) group (B). (C) Representative cranial healing 3D reconstruction images of the positive group (a), the negative group (b), and the WO group (c). (D) Data analysis graphs. Longer diameter of scab (a), time points indicate post-initial operation for the negative group and splint group, during post-secondary operation for the WO group. Scalp complete healing time (b). Relative cranial defect area ratio (RCDAR)=(Area at each timepoint/Area at 0 week)×100% (c). **P<0.01, compared with that of the negative group.

Scalp wound healing conditions are shown in Figure 2A, B. Compared to the negative group, wound healing of the silicone group was only delayed by about 6 days. In contrast, the wound obturator can be maintained for over 5 weeks. Additionally, after the removal of the obturator, wound healing duration was extended to 26.67±4.04 days, which is ~15 days longer than the non-splinted control group, fitting the criteria of a non-healing, chronic wound. Micro-CT scans and subsequent 3D reconstructions revealed distinct cranial bone healing patterns among the groups (Fig. 2C). In contrast to the positive control group, the negative control group demonstrated markedly slower healing, showing an RCDAR of 0.64±0.17, 0.53±0.11, and 0.43±0.11 at week 3, week 5, and week 12, respectively. Similarly, the WO group paralleled the negative control (Fig. 2D). These findings underscore the limited self-healing capacity of 6 mm cranial defects in rats without treatment over 12 weeks.

**Figure 3:**
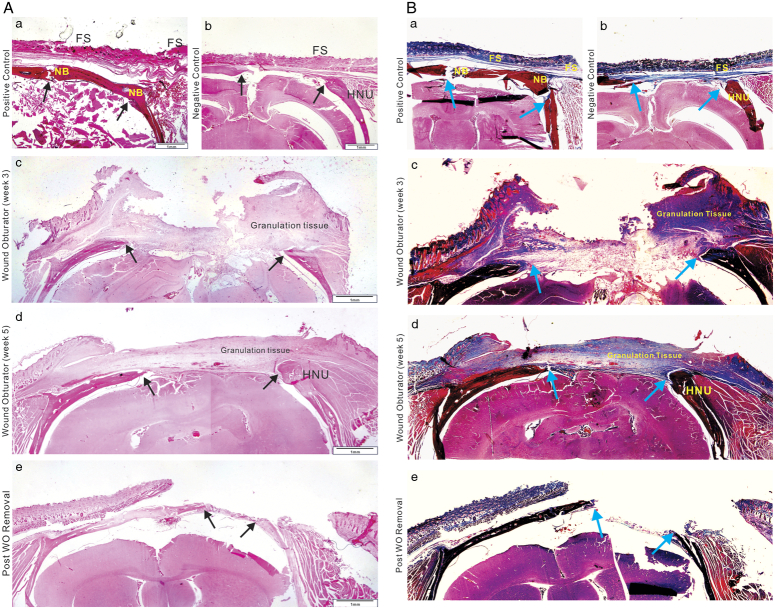
Histological staining of full-layer specimens. (A) H&E staining. (B) Trichrome staining. Full-layer specimens from positive control (*a*) and negative control (*b*) groups were collected at 5 weeks, WO group (week 3) (*c*), WO group (week 5) (*d*), and post WO removal (*e*) groups were fixed in 10% buffered formalin for 2 days, and decalcified in 5% nitric acid, followed by dehydration, paraffin embedding, and sectioning. Representative H&E staining results are shown. FS, fibrous scar; HNU, hypertrophic nonunion; NB, new bone.

Histological survey (Fig. 3) revealed that in the negative control group, the fibrous scar tissue significantly contracted into a smaller structure. For the wound obturator group at 3 weeks, the obturator had been freshly removed, thus presenting a chaotic histological phenotype, characterized by irregular fibrous scar and granulation tissue. By 5 weeks, however, the tissue appeared more organized and anatomically defined. The scalp defect was covered by granulation and fibrous tissue, with skin progressively enveloping this tissue, culminating in healing marked by a smaller wound. The bony margins of the osseous defects in the negative group expanded into a round shape reminiscent of ‘hypertrophic nonunion’ in clinical scenarios, thereby delaying or inhibiting the self-healing process^7^. In the WO group, the hypertrophic bone margin was not fully evident at 3 weeks post-trauma but became pronounced and consistent with that of the negative group by 12 weeks, suggesting that the first 3–4 weeks post-trauma may be critical for rapid bone healing.

## Discussion

Existing models for chronic skin defects in rodents include (1) Pressure ischemia–reperfusion, which involves multiple cycles of ischemia and reperfusion using a steel plate and magnet^8^. This method is cumbersome and the metal components interfere with micro-CT scans for studies involving cranial defects; (2) Diabetic rodents, which require a prolonged period to develop the diabetic condition and do not accurately replicate clinical scenarios^9^; and (3) Infection-based models, which are difficult to control and may adversely affect regenerative outcomes^10^. None of these models are ideally suited for studying composite cranial defects in rodents. Our approach employs mechanical resistance to counteract wound centripetal contraction. Compared to previously described methods, our method is simpler to implement, more closely aligns with trauma-related composite defects, and effectively extends the duration of a non-healing wound.

## Conclusion

We have successfully established a rat model of a scalp–cranial composite defect along with a unique complementary positive control, providing a stable platform for subsequent research. The experimental model effectively slowed the scalp healing process and preserved the cranial defect, embodying the characteristics of a ‘chronic composite defect.’ The positive control exhibited reliable and reproducible healing through autologous reconstruction, mirroring current surgical practice.

## Ethical approval

The protocols of this study were approved by the Institutional Animal Care and Use Committee (IACUC) of The University of Chicago (ACUP #71445).

## Consent

Not applicable.

## Source of funding

The reported work was supported in part by research grants from the National Institutes of Health (CA226303 to TCH and DE030480 to RRR). This project was also supported in part by The University of Chicago Cancer Center Support Grant (P30CA014599) and the National Center for Advancing Translational Sciences (NCATS) of the National Institutes of Health through Grant Number 5UL1TR002389. TCH was supported by the Mabel Green Myers Research Endowment Fund and The University of Chicago Orthopaedics Alumni Fund. Funding sources were not involved in the study design; in the collection, analysis, and interpretation of data; in the writing of the report; and in the decision to submit the paper for publication.

## Author contribution

R.R.R., L.W., Y.Z., T.-C.H., G.A.A., and C.S.: conceived and designed the study; Y.Z., O.M., H.Z., W.Y., and J.Z.: performed the experiments and collected data; Y.Z. and T.-C.H.: performed the statistical analysis; C.P.C., G.S., C.L., X.W., J.L., Y.S., Y.W., and J.F.: participated in experiments, provided essential experimental materials; and/or assisted in data analysis and interpretations; Y.Z., R.R.R., T.-C.H., G.A.A., S.C., H.H.L., L.L.S., and L.W.: drafted and revised the manuscript. All authors reviewed and approved the manuscript.

## Conflicts of interest disclosure

The authors declare no conflict of interest.

## Research registration unique identifying number (UIN)

Not applicable.

## Guarantor

Y.Z., T.-C.H., R.R.R., and G.A.A.

## Data availability statement

All data are available upon reasonable request.

## Provenance and peer review

Our reported work has not been submitted to or is not under consideration for publication by any other primary scientific journal.
